# The PDZ Domain of the Guanine Nucleotide Exchange Factor PDZGEF Directs Binding to Phosphatidic Acid during Brush Border Formation

**DOI:** 10.1371/journal.pone.0098253

**Published:** 2014-05-23

**Authors:** Sarah V. Consonni, Patricia M. Brouwer, Eleonora S. van Slobbe, Johannes L. Bos

**Affiliations:** Department of Molecular Cancer Research, Center for Molecular Medicine, University Medical Center Utrecht, Utrecht, The Netherlands; Karolinska Institutet Sweden

## Abstract

PDZGEF is a guanine nucleotide exchange factor for the small G protein Rap. It was recently found that PDZGEF contributes to establishment of intestinal epithelial polarity downstream of the kinase Lkb1. By binding to phosphatidic acid enriched at the apical membrane, PDZGEF locally activates Rap2a resulting in induction of brush border formation via a pathway that includes the polarity players TNIK, Mst4 and Ezrin. Here we show that the PDZ domain of PDZGEF is essential and sufficient for targeting PDZGEF to the apical membrane of polarized intestinal epithelial cells. Inhibition of PLD and consequently production of phosphatidic acid inhibitis targeting of PDZGEF to the plasma membrane. Furthermore, localization requires specific positively charged residues within the PDZ domain. We conclude that local accumulation of PDZGEF at the apical membrane during establishment of epithelial polarity is mediated by electrostatic interactions between positively charged side chains in the PDZ domain and negatively charged phosphatidic acid.

## Introduction

**Figure 1:**
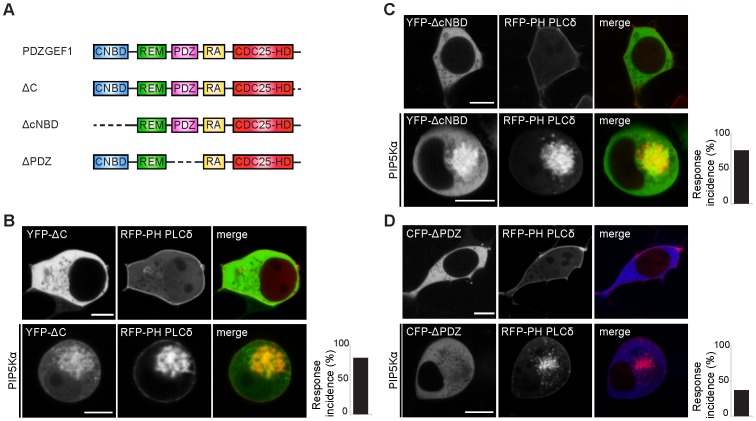
Characterization of the domain repsonsible for binding to phosphatidic acid. A. Domain architecture of PDZGEF mutants. cNBD: cyclic nucleotide binding domain; REM: Rap/Ras exchange motif; PDZ: post synaptic density protein (PSD95), Drosophila disc large tumor suppressor (Dlg1), Zonula occludens-1 protein (Zo-1); RA: Ras association domain; CDC25-HD: CDC25 homology domain. B, C. Mutants of PDZGEF either lacking the C-terminus (YFP-ΔC) or the cNBD domain (YFP-ΔCNBD) transfected in HEK293T cells are unable to associate with PA on vesicles in PIP5Kα-stimulated cells. RFP-PH PLCδ confirms production of PtdIns(4,5)P2 by PIP5Kα which then recruits the PA-producing enzyme PLD. D. Lack of the PDZ domain (CFP-ΔPDZ) abolishes association of the protein with PA on PIP5Kα-generated vesicles in HEK293T cells. The bar graphs show the percentage of cells showing vesicular localization of the protein in the presence of PIP5Kα (8/10 cells for YFP-ΔC, 9/12 cells for YFP-ΔCNBD and 4/11 cells for CFP-ΔPDZ). All scale bars: 10 µm.

The protein family of the Ras-like small GTPase Rap controls a variety of cellular pathways linked to cell adhesion, cell spreading and endothelial junction control [1]–[4]. Recently, an additional role for Rap in the regulation of brush border formation during intestinal epithelial polarity has been identified [5]. Rap proteins are kept under control by guanine nucleotide exchange factors (GEFs) that promote their active GTP-bound conformation and by GTPases accelerating proteins (GAPs) that induce their inactive state [6]. PDZGEFs are one of such Rap-specific exchange factors. They are characterized by the presence of a PDZ domain from which they derive their name and by a CDC25 homology domain (CDC25-HD) at their C-terminus present in all GEFs for Ras-like GTPases [7]. Moreover, they encompass one or two N-terminal cyclic nucleotide binding (cNBD) domains, even though they are unable to interact with cAMP or cGMP, a Rap/Ras exchange motif (REM) and Ras associating (RA) domain (Figure 1a) [8]. PDZGEFs have been recently identified as the GEFs responsible for the activation of Rap2a during brush border formation [5]. Establishment of intestinal epithelial polarity requires the formation of a polarization complex composed of the protein kinase Lkb1, the pseudo-kinase STRADα and the adaptor protein Mo25 [9], [10]. This results in establishment of apico-basal polarity and asymmetric distribution of polarity landmarks, such as accumulation of PtdIns(4,5)P2 at the apical membrane. Subsequent localization of phospholipase D (PLD) leads to local generation of phosphatidic acid (PA) and recruitment of PDZGEF. The following signaling cascade leads to Rap2a-mediated brush border formation [5].

Here we investigate which domain of PDZGEF is required for the apical recruitment of PDZGEF by PA during epithelial cell polarization. We find that the PDZ domain mediates apical localization of PDZGEF by directly binding to PA at the membrane. Moreover, we show that basic residues within the PDZ domain are needed for this interaction.

## Materials and Methods

### Antibodies, DNA constructs, cell culture and transfections

The anti-HA (12CA5) antibody was produced in-house. PDZGEF1 (RapGEF2) and PDZGEF2 (RapGEF6) were cloned C-terminal to a CFP or Citrine YFP tag in a pcDNA3 vector or an HA tag in a pmt2 vector using the Gateway system (Invitrogen). All mutants were generated by site-directed mutagenesis. Ls174T-W4 cells were mainted in RPMI medium supplemented with 10% tetracycline free fetal bovine serum (FBS), 100 U/ml penicillin and 100 µg/ml streptomycin (Lonza). HEK293T cells were maintained in Dulbecco's modified Eagle's medium (DMEM) supplemented with 10% fetal bovine serum (FBS), 100 U/ml penicillin, 100 µg/ml streptomycin and 1.4 mM L-glutamine (all from Lonza). Cells were transfected with expression plasmids using Xtreme gene 9 (Roche).

### Fluorescence microscopy

For confocal live-imaging, 1 day after transfection cells were seeded overnight in glass-bottomwells (WillCo Wells) and stimulated overnight with doxycycline (1 µg/ml) or treated with the PLD1 inhibitor CAY10593 (1 µM) (Cayman Chemicals) as indicated and examined in L-15 Leibovitz medium (Invitrogen) at 37°C. Images were acquired on an inverted Zeiss LSM510 confocal microscope equipped with 63× magnification objective lens (N.A. 1.4; Leica).

### Protein–Lipid Overlay Assays

HEK293T cells were transfected with HA tagged PDZ domain of PDZGEF and then lysed in buffer containing 50 mM Tris·HCl (pH 7.5), 150 mM NaCl, 1% Triton X-100, 2 mM MgCl2, and protease and phosphatase inhibitors. Lysates were then incubated overnight with protein G Sepharose beads and anti-HA antibody. The bound proteins were eluted with 3× HA pepetide (250 µg/mL) in BC300 buffer [20 mM Tris·HCl (pH 7.9), 20% glycerol, and 300 mM KCl] after washing, and protein recovery was determined by Western blotting. Eluted proteins were incubated with nitrocellulose membranes spotted with a variety of lipids as per manufacturer's instructions (PIP strips; Echelon Biosciences). Bound protein was detected using anti-HA antibody and visualized by Odyssey Infrared Imaging (Li-Cor).

## Results

### The PDZ domain of PDZGEF binds to phosphatidic acid

**Figure 2:**
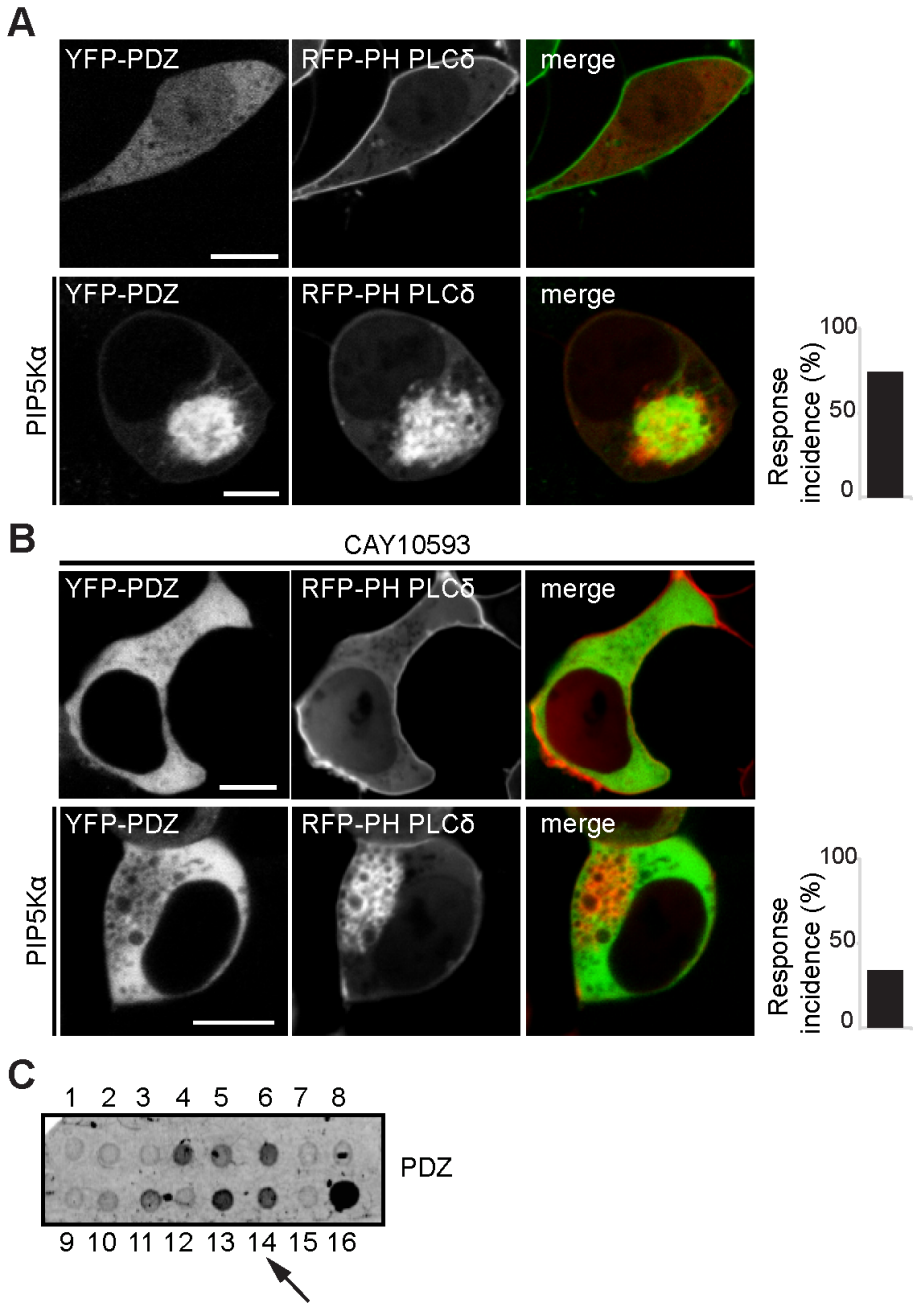
The PDZ domain of PDZGEF binds to phosphatidic acid. A. The PDZ domain of PDZGEF (CFP-PDZ) in HEK293T cells shows vesicular localization in the presence of PIP5Kα. B. Treatment of HEK293T cells with the PLD1 inhibitor CAY10593 impairs association of the PDZ domain (CFP-PDZ) with locally generated PA on PIP5Kα–generated vesicles. The bar graphs show the percentage of cells showing vesicular localization of the protein in the presence of PIP5Kα (8/11 cells for CFP-PDZ and 4/12 cells for CFP-PDZ in presence of CAY10593). All scale bars: 10 µm. C. Protein-lipid overlay assay of HA tagged PDZ domain of PDZGEF1 (PDZ) isolated from HEK293T cells using PIP strips containing 100pmol/spot of the following lipids: 1: lysophosphatidic acid (LPA), 2: lysophosphocholine (LPC), 3: phosphatidylinositol (PI), 4: phosphatidylinositol 3-phosphate [PI(3)P], 5: PI(4)P, 6: phosphatidylinositol 5-phosphate [PI(5)P], 7: phosphatidylethanolamine (PE), 8: phosphatidylcholine (PC), 9: sphingosine 1-phosphate (S1P), 10: phosphatidylinositol 3,4-bisphosphate [PI(3,4)P2], 11: phosphatidylinositol 3,5-bisphosphate [PI(3,5)P2], 12: phosphatidylinositol 1,2-bisphosphate [PI(4,5)P2], 13: phosphatidylinositol 3,4,5-trisphosphate [PI(3,4,5)P3], 14: phosphatidic acid (PA), 15: phosphatidylserine (PS) or 16: blue bank. Arrow indicates binding to PA.

To visualize association of PDZGEF with PA, we employed a previously used assay in HEK293T cells, as in these cell line it is possible to easily visualize association of a protein to phospholipids [5]. Briefly, HEK293T cells were transfected with the PtdIns(4,5)P2-producing enzyme PIP5Kα, which results in accumulation of PtdIns(4,5)P2 on endocytic vesicles. Such confined production of PtdIns(4,5)P2 can be readily visualized with the RFP-tagged PH domain of PLCδ [11]. The subsequent vesicular recruitment of PLD results in a local increase of PA and as a consequence accumulation of PDZGEF [5], [12], [13]. To determine which domain is responsible for the interaction of PDZGEF1 with PA, we analyzed a series of deletion mutants (Figure 1a). Of the mutants tested, only a PDZGEF1 mutant lacking its PDZ domain (ΔPDZ) was unable to accumulate at PA-enriched vesicles in PIP5Kα–stimulated cells (Figure 1b-d), suggesting that the PDZ domain is responsible for the interaction. Indeed, the PDZ domain of PDZGEF1 (PDZ) localizes at the endocytic vesicles of PIP5Kα- transfected cells (Figure 2a). We next investigated whether PA was indeed responsible for this interaction as previously observed for the full length PDZGEF protein [5]. We therefore treated the cells with CAY10593, an inhibitor of the PA-generating enzyme PLD1. We observed that the interaction between the PDZ domain of PDZGEF and PA was abolished (Figure 2b). This indicates that the PDZ domain interacts with PA. To confirm that the PDZ domain directly binds to PA, we carried out protein-lipid overlay assays. For this, several lipids spotted onto nitrocellulose membrane were incubated with HA tagged PDZ domain of PDZGEF1 isolated from HEK293T cells. Indeed, we detected binding of the PDZ domain to PA as well as to several other negatively charged lipids (Figure 2c). Similar results were obtained when the PDZ domain of the related family member PDZGEF2 was used (Figure S1). Although true specificity is lacking, these results show that the PDZ domain of PDZGEF can bind to PA *in vitro*.

### Basic residues within the PDZ domain are required for binding to PA

**Figure 3:**
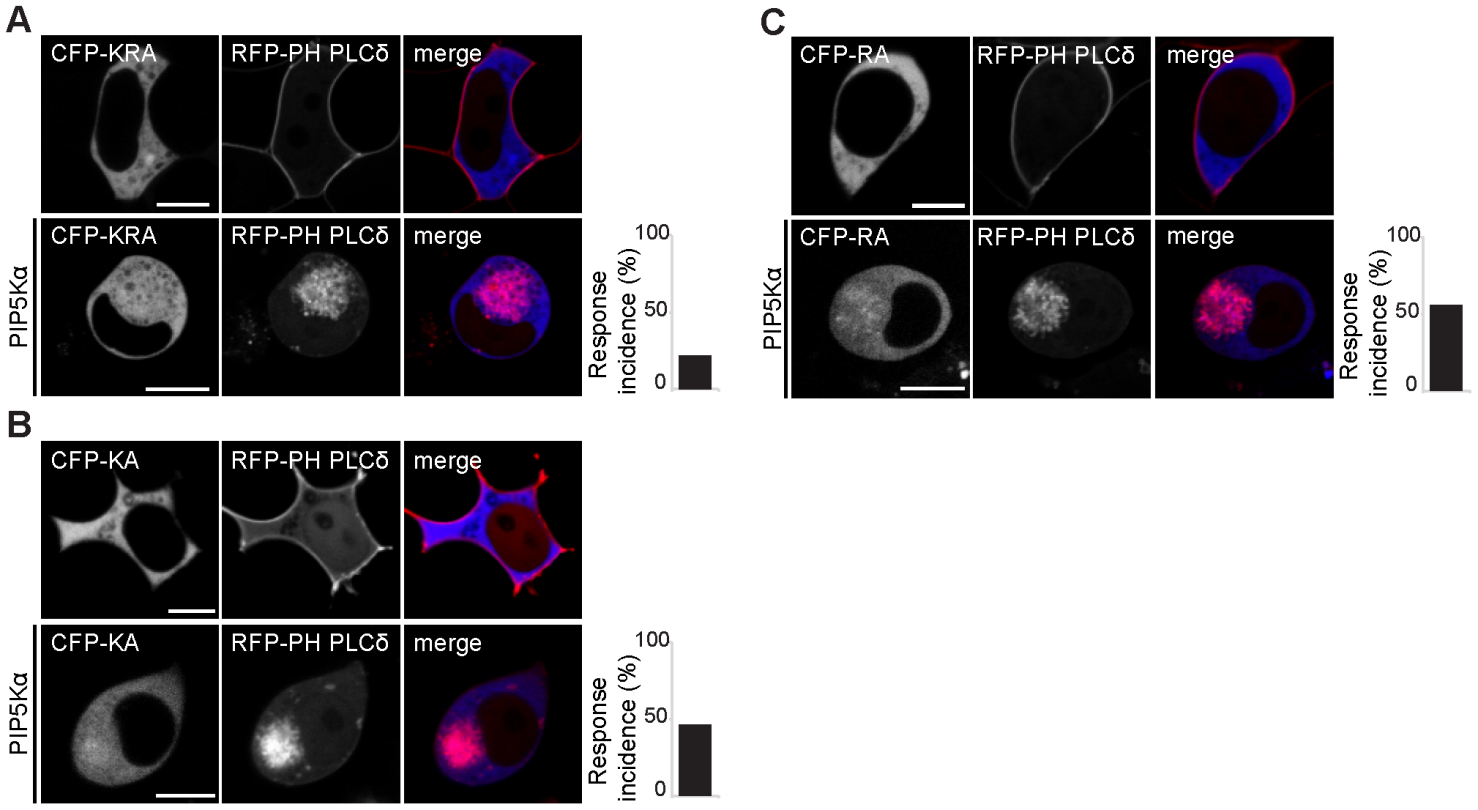
Positive residues within the PDZ domain are required for binding to PA. A. Mutation of lysine 428 and arginine 429 within the PDZ domain to a non-polar alanine (CFP-KRA) impairs interaction with PA on PIP5Kα-generated vesicles in HEK293T cells. B, C. Substitution of either lysine 428 or arginine 429 alone with alanine (CFP-KA and CFP-RA respectively) results in impaired ability of the protein to associate with vesicular PA in HEK293T cells. The bar graphs show percentage of cells localized on PIP5Kα-generated vesicles (3/11 cells for CFP-KRA, 5/11 cells for CFP-KA and 6/11 cells for CFP-RA). All scale bars: 10 µm.

Since positively charged residues are able to interact specifically with negatively charged phospholipids such as PA at the membrane, we next investigated whether basic residues within the PDZ domain contribute to apical membrane targeting of PDZGEF [14]. The PDZ domain of PDZGEF contains few positively charged amino acids, with lysine at position 428 (K428) and arginine at position 429 (R429) being the only basic residues clustered together. Mutational analysis revealed that the positively charged K428 and R429 are needed for binding to PA. Indeed, mutation of both of these residues to a non-polar alanine (KRA) abolished accumulation of PDZGEF1 to PA-enriched vesicles in PIP5Kα-stimulated cells (Figure 3a).

In order to determine which positively charged side chain contributes the most to the interaction with PA, single residue mutants were analyzed. Replacement of lysine 428 with an alanine (KA) decreased the ability of PDZGEF to interact with PA on endocytic vesicles (Figure 3b). Likewise, mutation of arginine 429 to alanine (RA) inhibited in part the localization of PDZGEF1 to PIP5Kα-generated PA-rich vesicles (Figure 3c).

The above results suggest that the positively charged lysine 428 and arginine 429 contribute to the apical accumulation of PDZGEF, but both residues are needed for protein localization and function.

### The PDZ domain localizes PDZGEF at the apical membrane during brush border formation via electrostatic interactions

**Figure 4:**
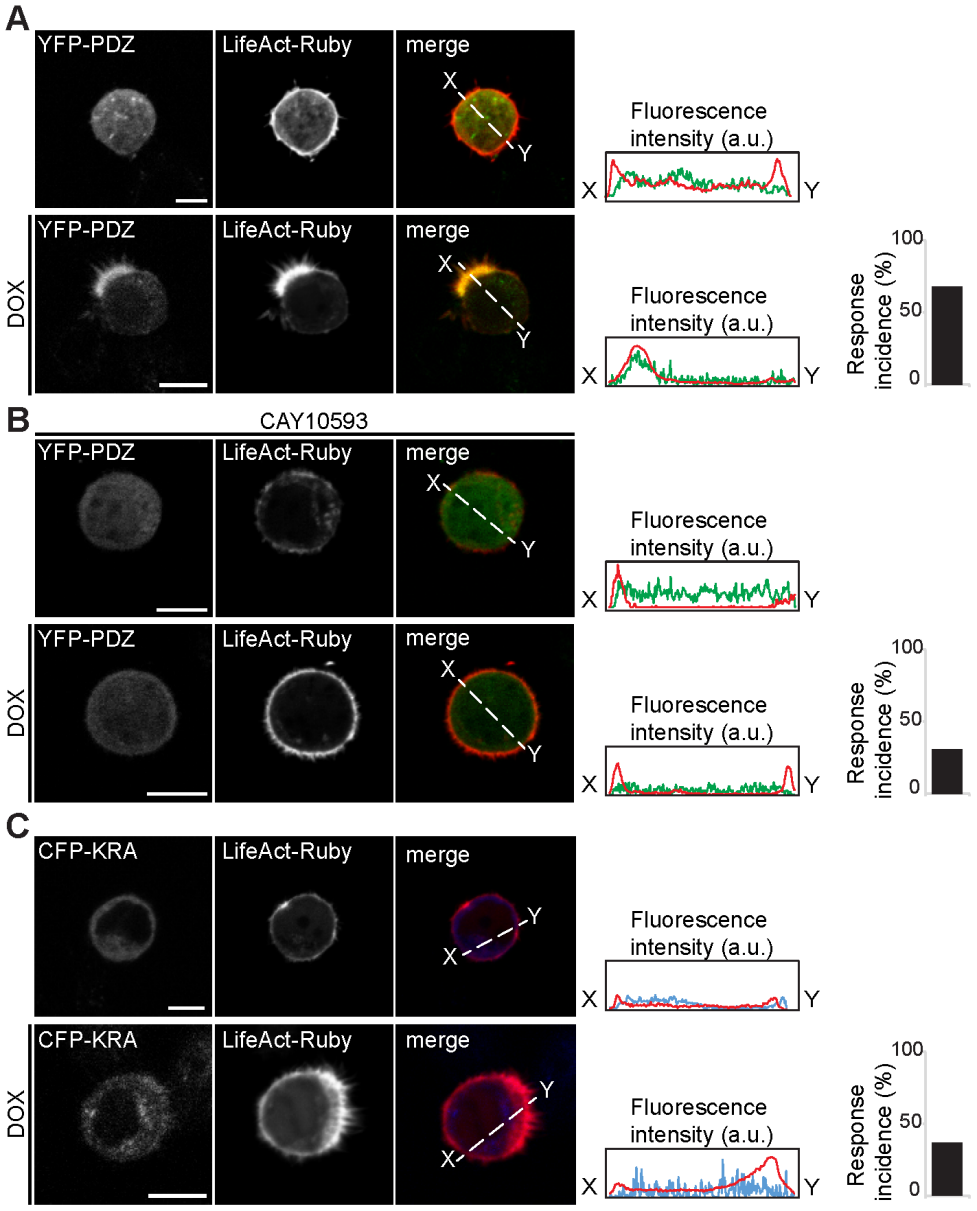
The PDZ domain localizes PDZGEF at the apical membrane during brush border formation via electrostatic interactions. A. Live-cell imaging of Ls174T-W4 cells transfected with YFP-PDZ and the actin binding probe LifeAct-Ruby with and without stimulation with doxycycline. B. Ls174T-W4 cells treated with the PLD1 inhibitor CAY10593 do not show accumulation of YFP-PDZ to the brush border of doxycycline-stimulated cells. C. The double mutant CFP- KRA is not able to localize at the brush border of Ls174T-W4 cells after doxycycline treatment. The profiles represent the fluorescence intensity across the cell as indicated by the dotted line. The bar graphs show the percentage of cells showing brush border localization of the protein following doxycycline treatment (8/12 cells for YFP-PDZ, 3/10 cells for YFP-PDZ in presence of CAY10593 and 4/11 cells for CFP-KRA). All scale bars: 10 µm.

We next validated the role of the PDZ domain in membrane targeting of PDZGEF during establishment of epithelial polarity. For this, we examined the localization of the PDZ domain in polarized cells. Ls174T-W4 intestinal epithelial cells were used for their ability to form a brush border upon doxycycline stimulation, which results in activation of the pseudo-kinase STRADα and subsequent establishment of polarity in individual cells by the kinase Lkb1 [10]. Live-imaging of single cells showed that, upon doxycycline stimulation, the PDZ domain of PDZGEF1 (YFP-PDZ) relocated to the brush border at the apical membrane, as also indicated by a similar accumulation pattern of the actin-binding probe LifeAct-Ruby (Figure 4a). Such apical redistribution in doxycycline stimulated cells was lost following treatment with the PLD1 inhibitor CAY10593 (Figure 4b), thus indicating that the PDZ domain interacts with PA at the brush border.

We next tested whether the positively charged amino acids within the PDZ domain also contribute to such polarized localization of PDZGEF. The KRA mutant was unable to accumulate to the brush border of doxycycline-stimulated cells (Figure 4c). Thus, the PDZ domain mediates localization of PDZGEF at the apical membrane by binding to PA via basic residues during intestinal epithelial polarity.

## Conclusions

We have recently shown that Rap2a activity is needed downstream of Lkb1 for induction of intestinal epithelial brush border formation via a pathway that employs its effector TNIK, the kinase Mst4 and Ezrin [5]. PDZGEF plays an important role in this as it mediates activation of Rap2a at the apical membrane. We find that the PDZ domain of PDZGEF directly binds to PA, enriched at the apical membrane during epithelial cell polarization. Moreover, we show that positively charged side chains within the PDZ domain are involved in such interaction, since their mutation to a non-polar amino acid abolishes apical localization of PDZGEF. Thus, this study provides further insights into the mechanisms by which PDZ domains mediate protein-lipid interactions.

PDZ domains usually interact with short motifs at the C-terminus of proteins [15]. Phosphorylation events or disulphide bonds formation contribute to the specificity of PDZ-mediated interactions [16]–[18]. The importance of PDZ domains as lipid-binding in addition to protein-binding modules in cellular signaling has recently become more and more appreciated. Indeed, PDZ domains have been shown to regulate a variety of cellular processes by binding mainly to the signaling lipid phosphatidylinositol [19]–[21]. A recent study also demonstrated that the PDZ domains of the *Drosophila* Par-3 homolog Bazooka directly bind to PA, and that this interaction influences establishment of polarity landmarks [22]. Our results demonstrate that PDZ domains can interact with PA also in mammalian cells.

## Supporting Information

Figure S1 **The PDZ domain of PDZGEF binds to phosphatidic acid.** A. In HEK293T cells PDZ domain of PDZGEF2 (CFP-PDZ2) localizes at vesicles in the presence of PIP5Kα. B. The PLD1 inhibitor CAY10593 impairs the ability of the PDZ domain of PDZGEF2 (CFP-PDZ2) to interact with PA on PIP5Kα–generated vesicles in HEK293T cells. The bar graphs show the percentage of cells showing vesicular localization of the protein in the presence of PIP5Kα (9/12 cells for CFP-PDZ2 and 3/11 cells for CFP-PDZ2 in presence of CAY10593). All scale bars: 10 µm. C. Protein-lipid overlay assay of HA tagged PDZ domain of PDZGEF2 (PDZ2) isolated from HEK293T cells using PIP strips containing 100pmol/spot of the following lipids: 1: lysophosphatidic acid (LPA), 2: lysophosphocholine (LPC), 3: phosphatidylinositol (PI), 4: phosphatidylinositol 3-phosphate [PI(3)P], 5: PI(4)P, 6: phosphatidylinositol 5-phosphate [PI(5)P], 7: phosphatidylethanolamine (PE), 8: phosphatidylcholine (PC), 9: sphingosine 1-phosphate (S1P), 10: phosphatidylinositol 3,4-bisphosphate [PI(3,4)P2], 11: phosphatidylinositol 3,5-bisphosphate [PI(3,5)P2], 12: phosphatidylinositol 1,2-bisphosphate [PI(4,5)P2], 13: phosphatidylinositol 3,4,5-trisphosphate [PI(3,4,5)P3], 14: phosphatidic acid (PA), 15: phosphatidylserine (PS) or 16: blue blank. Arrow indicates binding to PA.
(TIFF)
