# Supplementary figures and images for: The PDZ Domain of the Guanine Nucleotide Exchange Factor PDZGEF Directs Binding to Phosphatidic Acid during Brush Border Formation
Source: PLoS One. 2014 May 23;9(5):e98253. doi: 10.1371/journal.pone.0098253 (PMC4032295; doi:10.1371/journal.pone.0098253)

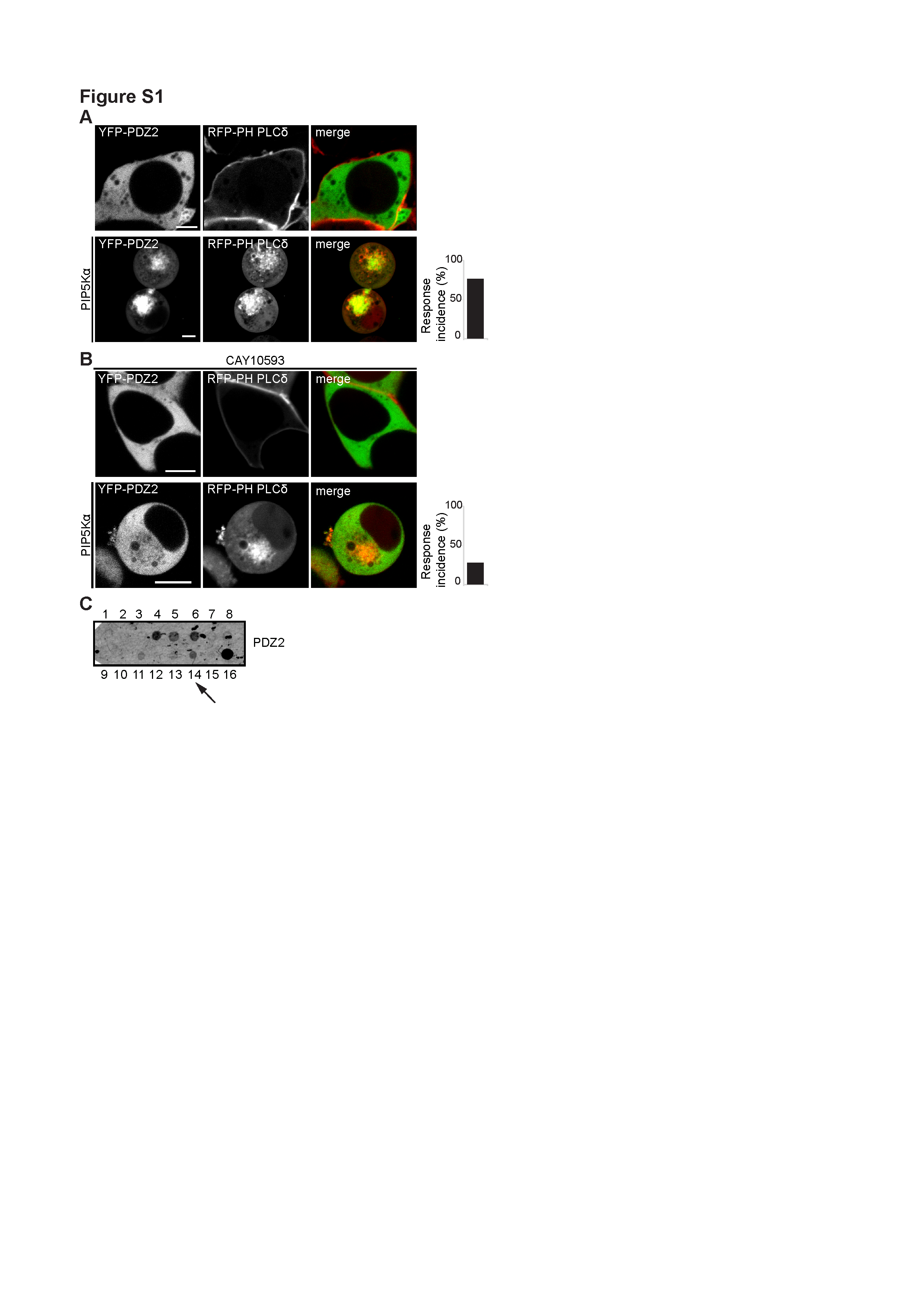

Supplement: Figure S1 — The PDZ domain of PDZGEF binds to phosphatidic acid. A. In HEK293T cells PDZ domain of PDZGEF2 (CFP-PDZ2) localizes at vesicles in the presence of PIP5Kα. B. The PLD1 inhibitor CAY10593 impairs the ability of the PDZ domain of PDZGEF2 (CFP-PDZ2) to interact with PA on PIP5Kα–generated vesicles in HEK293T cells. The bar graphs show the percentage of cells showing vesicular localization of the protein in the presence of PIP5Kα (9/12 cells for CFP-PDZ2 and 3/11 cells for CFP-PDZ2 in presence of CAY10593). All scale bars: 10 µm. C. Protein-lipid overlay assay of HA tagged PDZ domain of PDZGEF2 (PDZ2) isolated from HEK293T cells using PIP strips containing 100pmol/spot of the following lipids: 1: lysophosphatidic acid (LPA), 2: lysophosphocholine (LPC), 3: phosphatidylinositol (PI), 4: phosphatidylinositol 3-phosphate [PI(3)P], 5: PI(4)P, 6: phosphatidylinositol 5-phosphate [PI(5)P], 7: phosphatidylethanolamine (PE), 8: phosphatidylcholine (PC), 9: sphingosine 1-phosphate (S1P), 10: phosphatidylinositol 3,4-bisphosphate [PI(3,4)P2], 11: phosphatidylinositol 3,5-bisphosphate [PI(3,5)P2], 12: phosphatidylinositol 1,2-bisphosphate [PI(4,5)P2], 13: phosphatidylinositol 3,4,5-trisphosphate [PI(3,4,5)P3], 14: phosphatidic acid (PA), 15: phosphatidylserine (PS) or 16: blue blank. Arrow indicates binding to PA. (TIFF) [file pone.0098253.s001.tiff]
